# SMART MAT: Fibre Optic Innovation for Bedside Monitoring and Validation of Continuous Vital Signs

**DOI:** 10.3390/s25175321

**Published:** 2025-08-27

**Authors:** Jace L. A. Ng, Ahmad Azeem Bin Noordin, Clare W. M. Chan, Jael Chew, Clarissa W. Q. Lim, Nikhil T. Sanghavi, Omar Arif Bin Mohamed Murat, Hao Wen Tan, Lyn S. Yeo, Esther M. Y. Yow, Meredith T. Yeung

**Affiliations:** Health and Social Sciences Cluster, Singapore Institute of Technology, Singapore 828608, Singapore

**Keywords:** fibre optic technology, vital sign monitoring, continuous monitoring, remote monitoring, patient care, healthcare technology

## Abstract

Vital sign monitoring is fundamental to patient care. Although traditional intermittent systems are flawed, barriers to implementing continuous monitoring systems remain. The SMART MAT is a novel continuous monitoring device that detects vital signs remotely via fibre optic technology. The study aims to validate the SMART MAT and its paired devices against gold/clinical standard measurements for measuring heart rate (HR), respiratory rate (RR), systolic blood pressure (SBP), diastolic blood pressure (DBP), and oxygen saturation (SpO2). Healthy adults aged 21 to 80 were recruited for this cross-sectional study. Participants rested supine on a standardised mattress with the SMART MAT below. Vital signs were recorded over three five-minute intervals. Statistical analysis included descriptive statistics, two-way ANOVA, Mean Absolute Percentage Error (MAPE), and Bland–Altman plots. Among 321 participants recruited, HR and most RR measurements saw non-statistically significant differences (*p* > 0.05). Statistically significant differences were observed for SBP, SpO2, and most DBP measurements (*p* < 0.05). Only SBP measurements exceeded the acceptable limits of differences. Effect sizes were small to negligible (*n*^2^ < 0.04) and MAPE values were <20%. The SMART MAT has demonstrated reasonable accuracy and validity in monitoring vital signs in healthy adults. Alternative paired BP devices are recommended to enhance SBP measurement accuracy.

## 1. Introduction

Vital sign monitoring is essential for basic patient care and assessment. The integral role of measuring, trending, and interpreting vital signs for patient safety is well-documented in the literature. The five routinely recorded vital signs are heart/pulse rate (HR), respiratory rate (RR), blood pressure (BP), oxygen saturation (SpO2), and temperature [1,2]. Deranged vital signs promptly identify critical illnesses and clinical deterioration, allowing for timely intervention, improved patient outcomes, and reduced healthcare costs [1,2,3]. Vital sign monitoring is of paramount importance in the early identification of impending deterioration, as evidenced by the growing popularity, continuous development, and widespread adoption of physiological track and trigger systems in hospitals [4,5].

While the process of vital sign monitoring appears straightforward, traditional intermittent methods, which involve physically attaching devices and conducting periodic checks, are not devoid of criticism. Beyond their time-consuming nature, which diverts valuable time away from other essential tasks, inadequate staffing may result in inaccurate or incomplete sets of vital signs, thereby limiting the overall effectiveness of vital sign monitoring [6,7,8,9]. Clinical deterioration and delayed escalation of care may also result from infrequent, prolonged intervals between measurements, as well as a lack of expertise in interpreting changes in vital signs [1,8,10]. Additionally, traditional monitoring necessitates close interactions between healthcare personnel and patients, thereby potentially elevating the risk of disease transmission during health emergencies or pandemics [11]. Consequently, the advent of non-invasive, continuous, and remote vital sign monitoring systems, particularly those extending beyond traditional bedside and nursing station monitoring, has gained popularity as a solution to the above problems.

Over the last decade, technological advancements have seen the rise of wearable and contactless vital sign monitoring technologies. To illustrate, the Apple Watch, FitBit, and Garmin are some of the most well-known and widely commercialised wearable wrist monitors featuring photoplethysmography technology [12,13]. By leveraging light-emitting diodes to penetrate the skin and detect microvascular blood flow and volume changes through the wrist, these devices can measure pulse rate, a reliable proxy for heart rate in healthy individuals [12]. Various contactless monitoring systems employing different sensor technologies and their collective potential as an alternative to traditional monitoring methods have also been explored [14].

Fibre optic technology has garnered increasing recognition and emerged as a catalyst for innovation in continuous vital sign monitoring systems. Owing to its inherent immunity to electromagnetic interference (EMI), chemical inertness, biocompatibility, and small fibre dimensions, there has been a surge in the development of lightweight and mechanically robust sensors that are non-restrictive to movement in the form of wearable devices or contactless systems [15,16]. These notable advantages address key limitations of conventional electrical sensors for vital sign monitoring, including susceptibility to EMI, restricted mobility and discomfort from tethered setups, and limited accessibility due to costly, specialist-operated equipment [16,17].

A 2021 study has rigorously reviewed the current body of research on fibre optic sensor technology and recognised its potential to become prototypes for vital sign monitoring in healthcare settings [16]. Besides elaborating on the characteristics of the various fibre optic modalities employed in different technologies, the study also compiled lists of available studies that examined single and multi-parametric vital sign sensors. However, previous studies investigating the application of multi-parametric sensors in human subjects have been limited by suboptimal methodological quality. While most studies documented their novel fibre optic technology in technical detail, these studies are either limited by poorly defined research protocols that hinder reproducibility or by very small sample sizes, typically no more than ten participants, that restrict the generalisability of their findings to real-world applications [15,18,19,20,21,22,23,24,25,26]. Meanwhile, the study by Elsarnagawy et al. (2014) was conducted using simulations based on a specially designed algorithm rather than involving human subjects [27]. Other studies involve tightly adhered wearable sensors on various body parts (i.e., nose, wrist, chest), which compromises user comfort and practicality for daily wear and activities [15,19,20,24,25,26,28,29]. While the study by Yu et al. (2018) is noteworthy for its contactless set-up, it similarly lacked the methodological rigour necessary for large-scale implementation [30].

A technical explanation of the characteristics of different fibre optic sensors has also been narrated by Chen et al., (2014) [31]. To summarise the theoretical discourse on various fibre optic sensors, macrobending and hetero-core sensors lack the capability or sensitivity required for HR detection. Fibre Bragg grating (FBG) sensors enable simultaneous monitoring of HR and RR, but their dependence on complex and costly wavelength detection limits practicality. Other designs, such as statistical mode, higher-order mode, and interferometric sensors, are hindered by bulky hardware, high costs, or operational complexity. Plethysmography-based sensors lack integration as true sensing elements. Due to these limitations in design complexity, sensitivity, size, and cost, Chen et al., (2014) explored a multimode fibre optic technology that leverages microbending mechanisms for vital sign monitoring, proposed for clinical and home use [31]. This foundational work led to the development of the SMART MAT, intended for large-scale validation studies to evaluate the accuracy of the fibre optic technology before real-world implementation.

**Figure 1:**
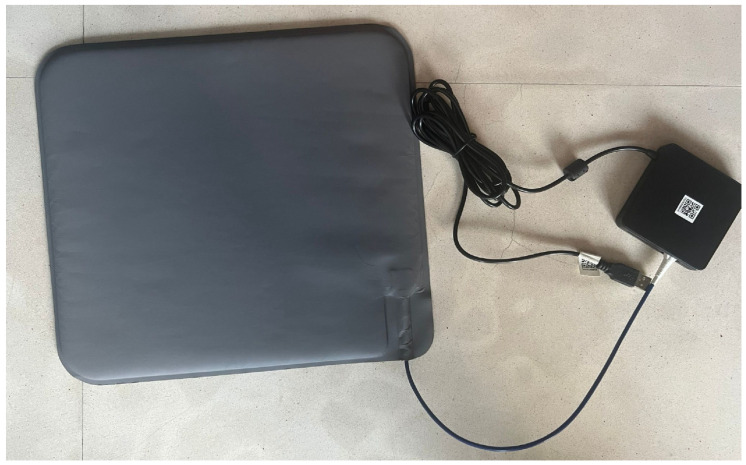
Form factors of the SMART MAT.

**Figure 2:**
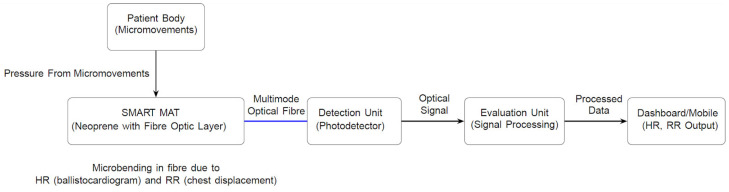
Working principle of the SMART MAT.

As depicted in Figure 1, the SMART MAT is a novel device featuring non-invasive, contactless, and continuous vital sign monitoring of HR and RR using multimode fibre optic technology (patent number 10201404690R), woven with a waterproof protective lamination. The fibre optic is embedded within a layered, mechanically structured sensor mat made from water-resistant neoprene. The SMART MAT system comprises a light source, a fibre coupler, a mirror, a photodetector, and a signal processing unit. Figure 2 illustrates the working principle of the SMART MAT. Micromovements in the body, specifically cardiac activity for HR and chest displacement for RR, elicit pressure-induced microbending in the fibre optic. The microbending effect is achieved by sandwiching the fibre between upper and lower layers of interwoven polyester fibre, functioning as a fibre mode converter to enhance mode coupling and stability. This configuration yields improved sensitivity and a more reliable signal-to-noise ratio. These changes then modulate light intensity, which is detected by the photodetector in the detection unit. The evaluation unit processes these signals to compute HR and RR remotely before displaying on the dashboard or linked devices.

The multi-channel ballistocardiogram (BCG) characterisation and algorithm (patent number 11201703737Q) is designed to capture and extract the recoil forces of the body resulting from cardiac ejection of blood into the vasculature, enabling identification of peak-to-peak intervals for HR calculation. To further enhance accuracy, the BCG applies cepstrum smoothing to optimise signal quality and refine HR detection. This approach enhances the estimation of HR by addressing noise, motion artefacts, and the non-stationary nature of BCG signals. The system digitises BCG signals from multiple fibre optic sensors and applies frequency-domain transformations to obtain smoothed cepstral representations. These are fused to derive a robust estimate of heartbeat intervals, with dynamic channel selection employed to optimise signal quality across varying conditions. Conversely, pressure variation analysis detects cyclic chest displacement signals via pressure changes, enabling determination of breath-to-breath intervals and RR.

Advanced signal processing techniques, including peak detection, Fast Fourier Transform (FFT), and wavelet analysis, are employed to extract physiological parameters from the modulated optical signals. The processed data are subsequently displayed and used to trigger alarms when pre-defined physiological thresholds are breached, enabling timely intervention and highlighting its potential for clinical use. Additionally, the non-wearable, compact design allows for seamless integration into smart beds or chairs and maximises user comfort.

The SMART system is also capable of integrating with additional paired devices to measure BP and SpO2 for comprehensive vital sign monitoring. This integration enhances its potential for use in community and routine care settings, supporting early detection and prompt intervention. Collectively, these innovations represent a significant advancement in fibre optic vital sign monitoring, offering a robust, low-cost, and non-invasive alternative to conventional techniques, with promising applications in clinical, residential, and wellness domains. To date, the SMART MAT has yet to undergo formal validation.

As the first large-scale validation study conducted on the SMART MAT, this research aims to validate its accuracy in measuring vital signs among healthy adults against the established gold/clinical standard measurements. As two models of the SMART MAT have been developed during the study period, this study will also contrast the findings obtained from both models to determine if they exhibit comparable accuracy and allow for collective testing against the gold/clinical standard measurements. The following gold/clinical standard measurements were selected for each vital sign:

(1) HR: gold standard measurement using an ECG [32],
(2) RR: clinical standard measurement by manual counting of RR over one minute at rest [33],
(3) BP: gold standard measurement using the auscultatory technique with a manual sphygmomanometer [34], and
(4) SpO2: clinical standard measurement via fingertip pulse oximeter [35].

It is hypothesised that the SMART MAT will demonstrate accuracy and validity comparable to the current gold/clinical standards. Additionally, both SMART MAT models will yield comparable levels of accuracy. Besides contributing to the ever-growing body of research on advanced vital sign monitoring technologies, this may also set a precedent for future validation studies in larger or more specialised populations, thereby supporting broader clinical adoption and enhancing healthcare productivity.

## 2. Materials and Methods

### 2.1. Research Design and Ethics

Following the recommendation of the STROBE guidelines [36], the cross-sectional study was conducted between April 2023 and September 2024 to evaluate the accuracy and validity of the vital signs measured by the SMART MAT against the established gold standard/clinical measurements. Data collection took place in the laboratory of the testing facilities. The University Institute of Review Board approved the research protocol (IRB number 2023021). Participants provided written informed consent after the researchers provided the study purpose, procedures, and protocol. Participation was voluntary, and participants were free to withdraw from the study at any time. Unique identification codes were assigned to all participants to ensure anonymity. All data collected was encrypted and stored on a cloud-based storage system, accessible only to the study investigators.

### 2.2. Participants

Participants were recruited via convenience sampling. Inclusion criteria were individuals aged 21 and above who were willing to participate in the study. Potential participants were excluded if they presented with the following:

(1) A history of mastectomy with radiation therapy with or without axillary lymph node dissection, due to the increased risk of lymphedema associated with BP measurement on the ipsilateral arm [37,38,39],
(2) Presence of arteriovenous fistula or dialysis shunt, as BP measurement on the ipsilateral arm also poses elevated risks of trauma and clot formation [40],
(3) A known allergy to the adhesive on the ECG, as a preventative measure against the onset of skin sensitivity or an allergic reaction [41], and
(4) A known history of irregular heart rhythms, including atrial fibrillation and atrial flutter, for arrhythmia is associated with highly inconsistent and variable ventricular rhythms that could lead to false positives in observed differences in HR [42,43].

### 2.3. Sample Size Calculation

The sample size was calculated based on preliminary data from the 50 paired pilot measurements comparing the SMART MAT to gold/clinical standard methods for HR, RR, SBP, DBP, and SpO2. The primary outcome was the mean difference in HR, estimated at 1 bpm with a standard deviation (SD) of 3.46 bpm, derived from the interquartile range of differences. A repeated-measures ANOVA was planned, requiring 160–179 subjects for 90% power at α = 0.01 (Bonferroni-corrected for five variables), assuming normality. To account for potential non-normality, for which ANOVA is robust at large sample sizes, and to ensure adequate power for secondary outcomes, particularly DBP (1.67 mmHg, requiring 309 subjects for 80% power), a sample size of 309 was selected. This provides >95% power for HR, >99% power for SBP (4.67 mmHg), 80% power for DBP, 45% power for RR (0.33 breaths/min) with >95% power for a clinically meaningful RR difference of 1 breath/min, and >99% power for SpO2 equivalence testing with a ±2% margin (α = 0.05). A sample of *n* = 309 is sufficient if non-normality persists. This sample size aligns with regulatory requirements for the validation of medical devices.

### 2.4. Instrumentation

#### 2.4.1. Anthropometric Measurements

The Tanita MC-980Uplus Multi Frequency Segmental Body Composition Analyser (Tanita Corporation, Tokyo, Japan) analysed the body composition with 40 measurements in under 30 s [44]. The Seca 213 Portable Stadiometer measured the height with a graduation of 1 mm [45].

#### 2.4.2. Gold/Clinical Standard Devices

The Schiller CardioVit AT-1 G2 Electrocardiogram (Schiller, Baar, Switzerland) recorded the HR, rhythm, and electrical activity [46]. Its outstanding signal quality in cardiac monitoring is recognised across both adult and paediatric populations.

The Bokang LCD Mercury-Free Sphygmomanometer (BK1016B) (Wenzhou Bokang Instrument, Co., Ltd. Wenzhou, China) measured BP manually through the pressurisation of the inflation bulb and controlled depressurisation via an air release valve [47]. Used in conjunction with a stethoscope, it allows for accurate BP measurements up to 300 mmHg.

The Nellcor™ Portable SpO2 Patient Monitoring System (PM10N) (Medtronic, Minneapolis, MN, USA) reported SpO2 levels non-invasively and is known to provide accurate readings even under challenging conditions such as low perfusion, signal interference, and patient motion [48].

#### 2.4.3. SMART MAT

The SMART MAT (780 mm × 780 mm × 6 mm), developed by Masstron Pte Ltd. (Singapore) under patented licenses owned by the Agency for Science, Technology and Research, harnesses fibre optic technology to remotely detect HR, RR, and bed occupancy. The detection unit houses a photodetector that captures changes in light intensity resulting from microbending in the optical fibre. The unit is sensitive to microbend-induced light loss correlated with physiological movements of the heart and chest, converting analogue optical signals into digital electrical signals for further processing by the evaluation unit. The evaluation unit, consisting of a compact processing module with a microprocessor and memory, implements proprietary algorithms for BCG and pressure variation analysis to isolate micromovements and determine HR and RR, respectively, in real time. A bandpass filter is also included to eliminate noise and isolate physiological signals.

**Figure 3:**
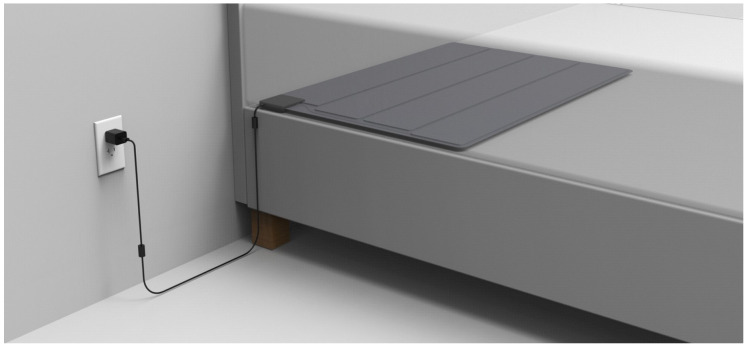
Application and set-up of the SMART MAT.

**Figure 4:**
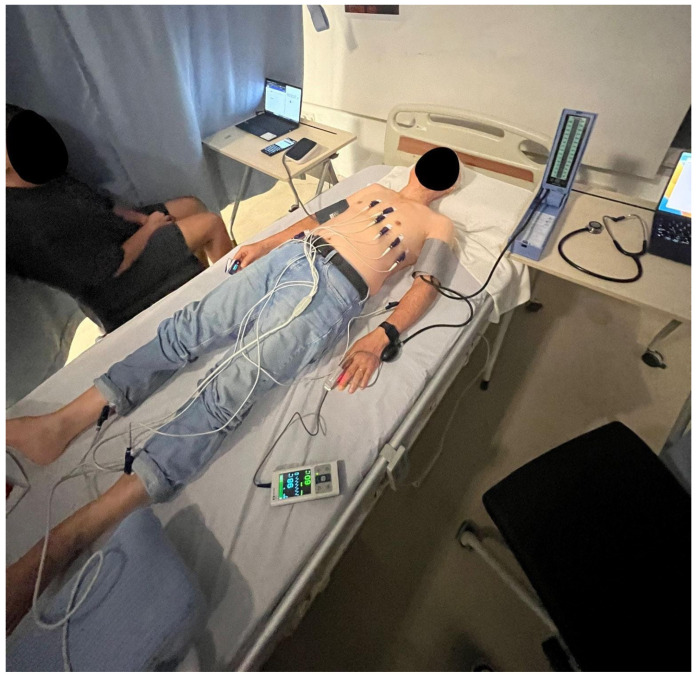
Actual set-up of the research protocol.

Figure 3 and Figure 4 depict the application of the SMART MAT and the actual set-up of the research protocol. As seen in Figure 4, the SMART MAT can be integrated with external peripheral devices to measure BP and SpO2 for completeness of vital signs measurement parameters. The evaluation unit aggregates all data, including BP and SpO2 from paired devices synchronised with HR and RR via timestamps, into a unified format for real-time monitoring. Remote, real-time vital sign monitoring by the SMART MAT is then achieved by transmitting data output from the evaluation unit via Wi-Fi or ethernet connectivity to a central dashboard or mobile devices.

Throughout this study, two models of the SMART MAT were subjected to identical testing procedures in comparison to established gold-standard clinical methods. As part of design optimisation for improved efficiency, the SMART MAT I, used in stage 1, incorporated a dual-channel optic fibre configuration, while the SMART MAT II, used in stage 2, adopted a single-channel configuration. Sensorgram plots are presented in Supplementary File S1, and the comparative analysis between the SMART MAT and the gold/clinical standard measurements is provided in Supplementary File S2.

#### 2.4.4. Paired SMART MAT Devices

The Jumper Electronic Blood Pressure Monitor (JPD-HA121) (Jumper, Shenzhen, China) conveniently measures BP non-invasively using an oscillometric technique, with automatic cuff inflation and deflation [49]. The device is registered with the Health Science Authority of Singapore (DE0507070) and has received both CE marking and United States Food and Drug Administration (FDA) approval.

The Jumper Pulse Oximeter (JPD-500F) (Jumper, Shenzhen, China) is a lightweight fingertip pulse oximeter designed for non-invasive monitoring of SpO2 using photoelectric oxyhaemoglobin inspection technology [50]. The device is registered with the Health Science Authority of Singapore (DE0507086) and has obtained both CE marking and FDA approval.

### 2.5. Research Protocol

Prior to each participant’s session, a set of pre-study instructions were delivered to ensure standardisation and accuracy of measurements. The instructions were as follows:

(1) Wear comfortable and loose clothing,
(2) Empty bladder and bowel as needed,
(3) Remove any nail polish,
(4) Refrain from applying skincare products on the body,
(5) Refrain from eating and drinking 30 min before the session,
(6) Refrain from vigorous activity 1 h before the session,
(7) Refrain from consuming caffeine and alcohol 24 h before the session,
(8) Refrain from diuretics within seven days of the session, and
(9) Inform co-investigators of the study if they are unwell or unable to attend the session.

Upon arrival at the study site, at least two co-investigators were present to proceed with the data collection. One co-investigator briefed each participant on the study procedure before obtaining written informed consent, while the second co-investigator measured the anthropometric data using the bioelectrical impedance analysis machine and stadiometer. Upon removing footwear and cleaning both feet with alcohol wipes, the following variables were recorded: age, gender, height, weight, body mass index, muscle mass, body fat percentage, and total body water percentage.

With reference to Figure 3, participants were instructed to lie supine on a bed with a standardised mattress height of 10 cm and the SMART MAT positioned below. The first co-investigator assisted in attaching the gold/clinical standard devices, including the ECG electrodes on the chest, the sphygmomanometer cuff, and the pulse oximeter on the left. The second co-investigator assisted in attaching the SMART MAT-paired devices, including the electronic blood pressure cuff and the pulse oximeter on the right. Before commencing the data collection, the SMART MAT was allowed approximately five minutes to calibrate. Participants were instructed to minimise movement and conversation throughout as such activities may compromise measurement accuracy by introducing motion artefacts or disrupting continuous signal acquisition.

Three data reading points for each vital sign were taken at five-minute intervals. HR and BP were measured at the 3rd, 8th, and 13th minute, while RR and SpO2 were measured at the 5th, 10th, and 15th minute. Each co-investigator was responsible for charting the vital signs measured using either the gold/clinical standard devices and methods or the SMART MAT and its paired devices separately. Before leaving the study site, participants were monitored for five minutes for any symptoms or adverse reactions to the study procedure. The entire research protocol took approximately 30 min per participant.

### 2.6. Statistical Analysis

The JASP software Version 0.19.3 was used to perform the statistical analysis. Descriptive statistics were used to determine the participants’ demographic properties. Subsequently, the Kolmogorov–Smirnov test was used to assess the parametric properties of the vital sign readings. Non-parametric statistical tests were selected for standardisation. The first two-way ANOVA and effect size ($n^{2}$) compared the data collected between the double-channel SMART MAT I and single-channel SMART MAT II; the second two-way ANOVA and $n^{2}$ determined statistical significance differences between the readings of the gold/clinical standard measurements and both SMART MAT devices. The interpretation of $n^{2}$ is as follows: 0.01 represents a small effect size, 0.06 is a medium effect size, and 0.14 is a large effect size [51]. The Mean Absolute Percentage Error (MAPE) quantified the accuracy of the SMART MAT using the percentage of discrepancy between its readings from the gold/clinical standard methods, where <10% indicates high accuracy, 10–20% indicates good accuracy, 20–50% is average accuracy, and >50% indicates inaccuracy [52]. Lastly, descriptive statistics were included to facilitate comparative analysis between the SMART MAT and the gold/clinical standard measurements. Bland–Altman plots were compiled to assess the agreement between the gold/clinical standard measurement and the SMART MAT [53]. All statistical analyses were performed at a 95% confidence level, with *p* < 0.05 considered statistically significant. Missing data was dealt with by excluding all data collected by the involved participant.

## 3. Results

### 3.1. Participant Characteristics

A total of 321 eligible participants completed the data collection, of which 120 participants used the SMART MAT I and 201 participants used the SMART MAT II. There were no withdrawals or missing data throughout the study. All data points were retained in the analysis; no outliers were removed. The baseline demographic and anthropometric data are presented in Table 1.

### 3.2. Two-Way ANOVA Between SMART MAT I and II

The results of the two-way ANOVA comparing the double-channel SMART MAT I and single-channel SMART MAT II are recorded in Table 2. Although the mean values of SBP, DBP, and RR appeared to differ between both models (F > 1), none of the vital signs demonstrated a statistically significant difference (*p* > 0.05). Additionally, the effect sizes were small to negligible ($n^{2}$ ≤ 0.01), suggesting that any observed differences lack clinical significance. Therefore, it is methodologically justifiable to pool data from both SMART MAT models to increase the sample size for a more robust statistical analysis.

### 3.3. Heart Rate

**Figure 5:**
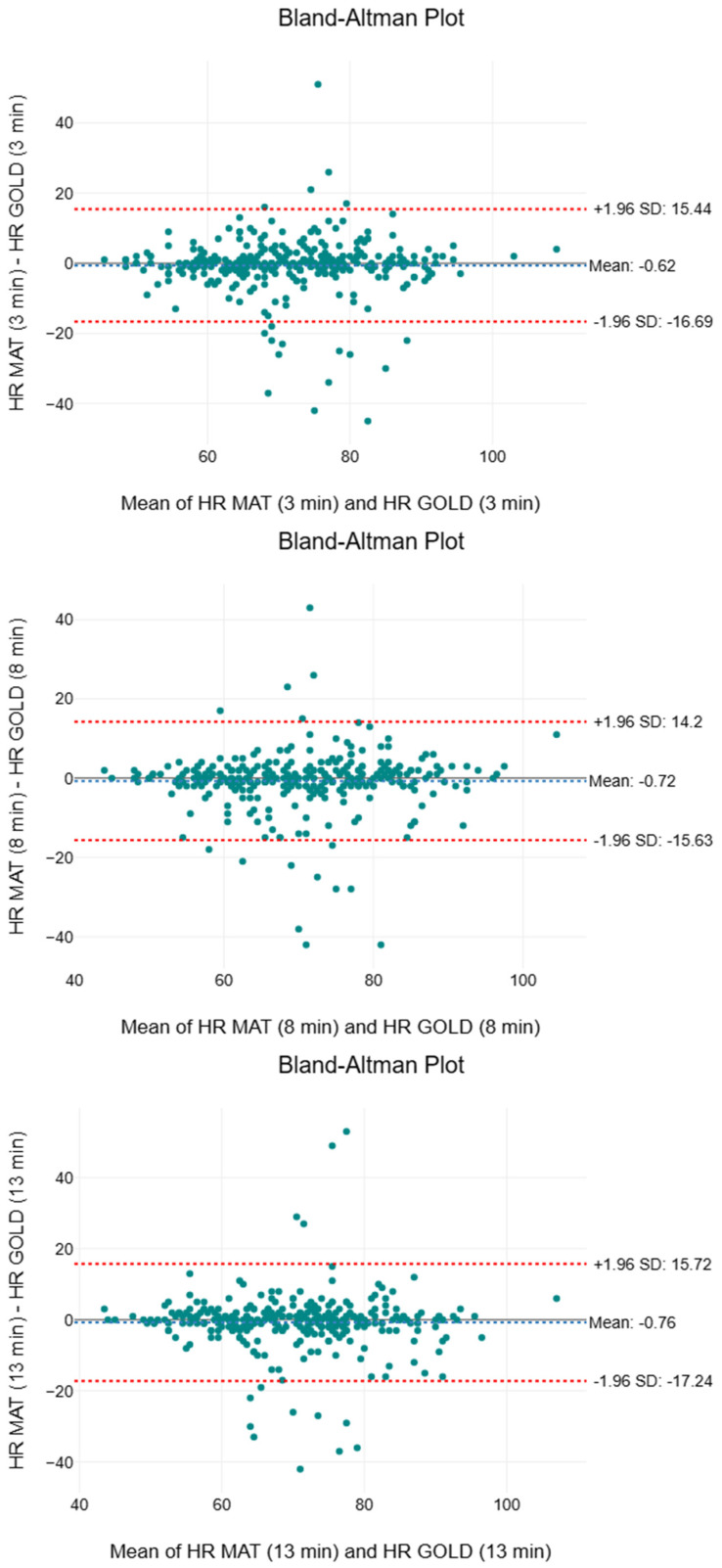
Compilation of Bland–Altman plots for heart rate.

Table 3 summarises the descriptive and inferential statistics, including the two-way ANOVA, effect sizes, and MAPE, for HR. The means and medians of both measurement methods consistently ranged between 70 to 71 beats per minute (beats/min). Although the first quartile values of the SMART MAT were consistently 1 to 2 beats/min lower than those of the gold standard (61 to 62 beats/min vs. 62 to 64 beats/min), the third quartile values of the two measurement methods were identical or overlapping. The F-values were 1.95, 3.02, and 2.75, respectively. No statistically significant differences (*p* > 0.05) between the two measurement methods with negligible effect sizes ($n^{2}$ < 0.01) were observed. The MAPE values were 6.45%, 6.24%, and 6.44%, respectively. Figure 5 shows a compilation of the Bland–Altman plots for HR. The mean bias ranged from −0.72 to −0.62 beats/min, indicating a very minor underestimation by the SMART MAT. The 95% limits of agreement spanned from −17.24 to 15.72 beats/min.

### 3.4. Respiratory Rate

**Figure 6:**
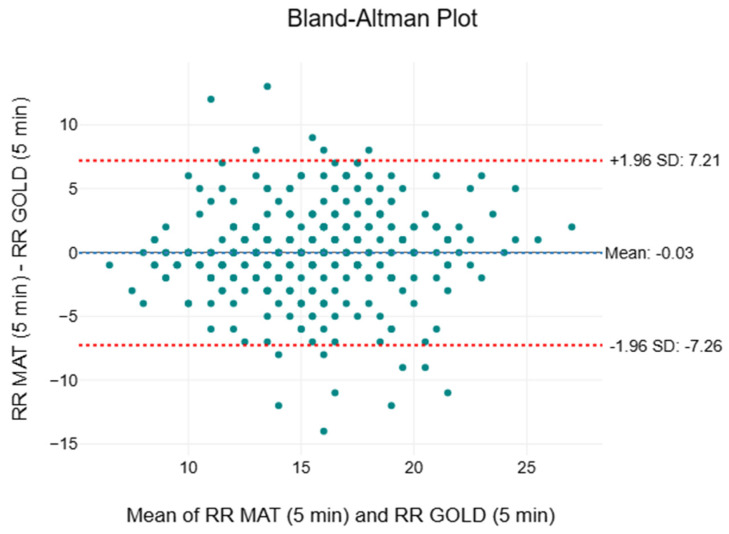

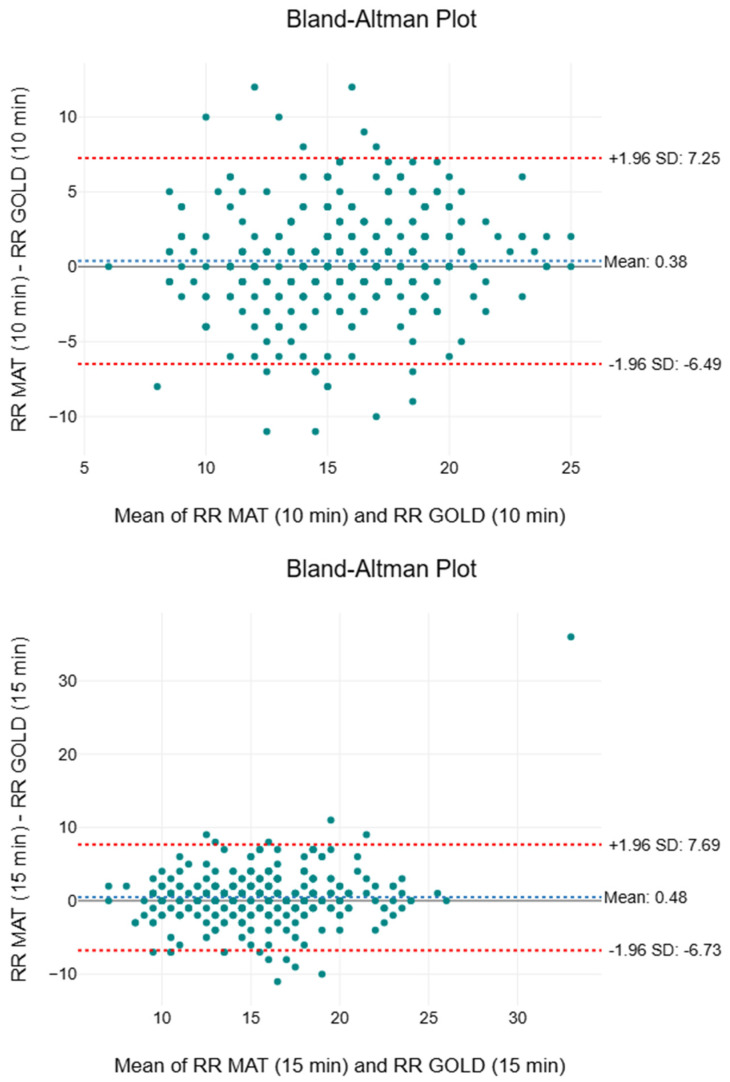
Compilation of Bland–Altman plots for respiratory rate.

Table 4 documents the descriptive and inferential statistics, including the two-way ANOVA, effect sizes, and MAPE, for RR. The means and medians of the clinical standard were consistent at 15 breaths per minute (breaths/min), while the means and medians of the SMART MAT fluctuated between 15 and 16 breaths/min. The interquartile ranges (IQR) of both measurement methods were identical throughout (IQR = 13 to 18). A progressive increase in F-values from 0.02 to 5.45 was noted. Only statistically significant differences (*p* = 0.020) were observed at the 15th minute. However, negligible effect sizes ($n^{2}$ < 0.01) were noted throughout. The MAPE values were 18.3%, 17.9%, and 15.3%, respectively. Figure 6 depicts a compilation of the Bland–Altman plots for RR. The mean bias ranged from −0.03 to 0.48 breaths/min, indicating an almost insignificant variation between the two measurement methods. The 95% limits of agreement spanned from −7.26 to 7.69 breaths/min.

### 3.5. Systolic Blood Pressure

**Figure 7:**
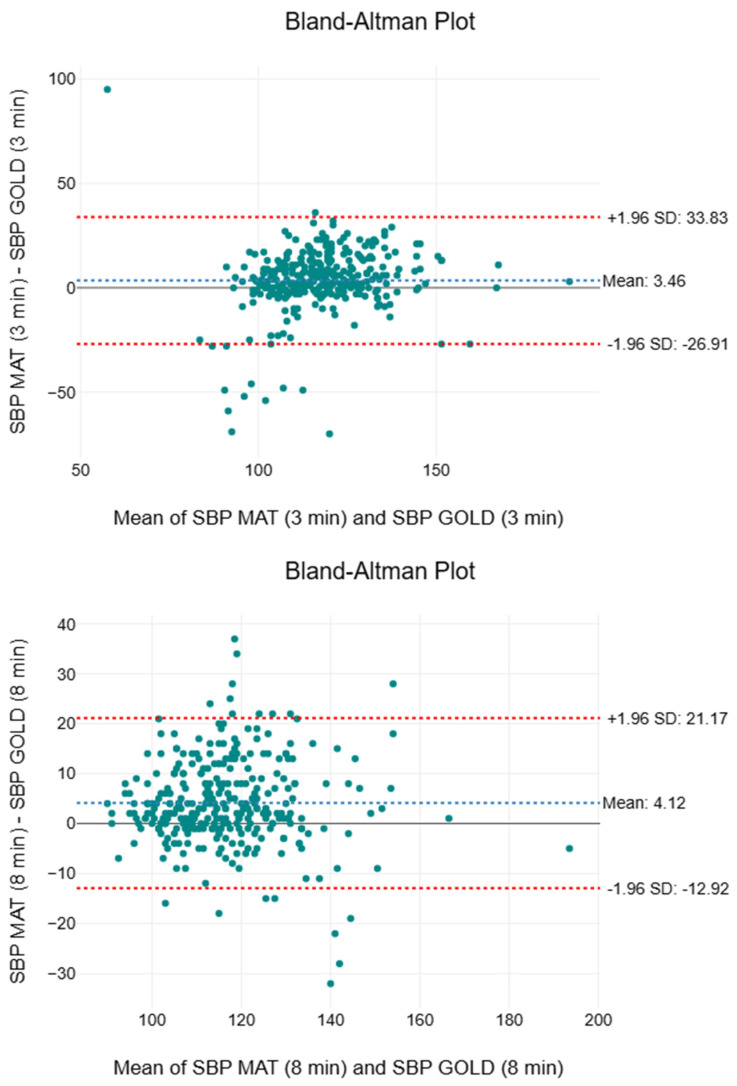

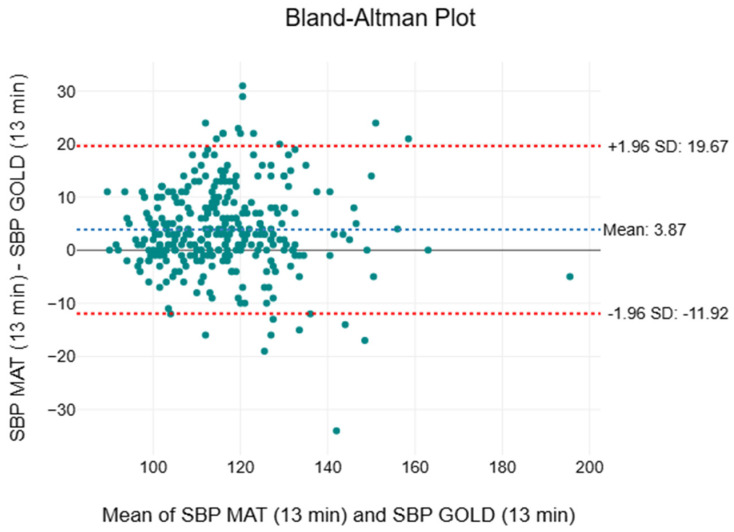
Compilation of Bland–Altman plots for systolic blood pressure.

Table 5 depicts the descriptive and inferential statistics, including the two-way ANOVA, effect sizes, and MAPE, for SBP. The medians recorded by the gold standard (111 to 113 mmHg) were consistently lower than those of the paired SMART MAT device (117 to 120 mmHg). This pattern was also observed in the first (103 to 106 mmHg vs. 107 to 110 mmHg) and third (121 to 123 mmHg vs. 125 to 131 mmHg) quartile values between the two measurement methods. Besides the notably high F-values of 87.7, 72.9, and 74.5, respectively, statistically significant differences (*p* < 0.001) were observed between the two measurement methods throughout. However, the effect sizes diminished over time from 0.04 at the 3rd minute to 0.02 at the 8th and 13th minutes. The MAPE values were 7.16%, 5.67%, and 5.39%, respectively. Figure 7 presents a compilation of the Bland–Altman plots for SBP. The mean bias ranged from 3.46 to 4.12 mmHg, indicating a consistent overestimation by the SMART MAT. The 95% limits of agreement spanned from −26.91 to 33.83 mmHg.

### 3.6. Diastolic Blood Pressure

**Figure 8:**
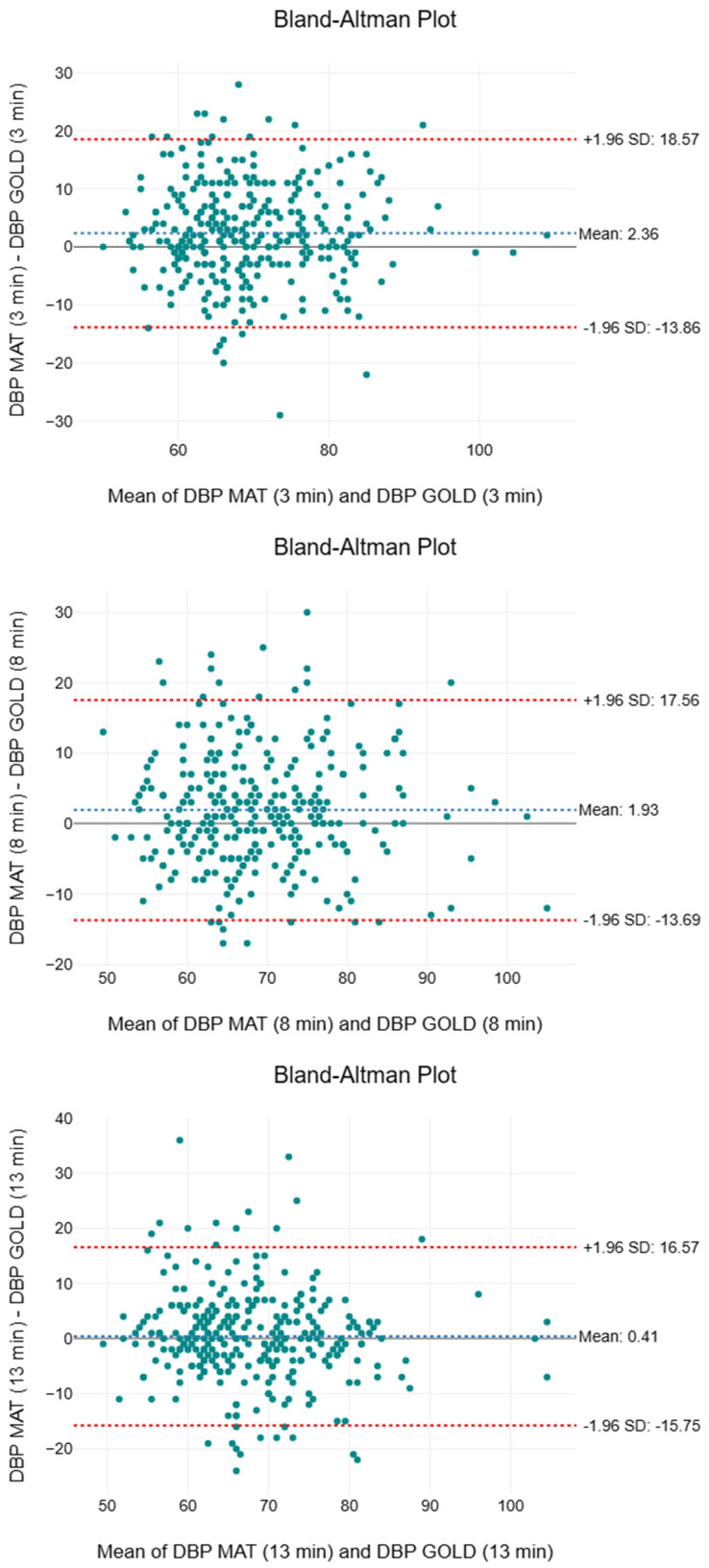
Compilation of Bland–Altman plots for diastolic blood pressure.

Table 6 presents the descriptive and inferential statistics, including the two-way ANOVA, effect sizes, and MAPE, for DBP. All the means are marginally higher than the medians by 1 to 2 mmHg. The medians recorded by the gold standard (66 to 67 mmHg) were marginally lower than that of the paired SMART MAT device (67 to 69 mmHg). Although the IQR overlapped, the third quartile values of the paired SMART MAT device (76 to 77 mmHg) were 2 to 3 mmHg higher than those of the gold standard (74 mmHg) at the 3rd and 8th minutes. The F-values showed a decreasing trend from 26.7 to 0.80. Statistically significant differences (*p* < 0.001) were observed between the two measurement methods only at the first two intervals. The effect sizes ($n^{2}$ ≤ 0.01) were consistently small to negligible throughout. The MAPE values were 9.22%, 8.76%, and 8.78%, respectively. Figure 8 displays a compilation of the Bland–Altman plots for DBP. The mean bias ranged from 0.41 to 2.36 mmHg, indicating a consistent overestimation by the SMART MAT. The 95% limits of agreement spanned from −15.75 to 18.57 mmHg.

### 3.7. Oxygen Saturation

**Figure 9:**
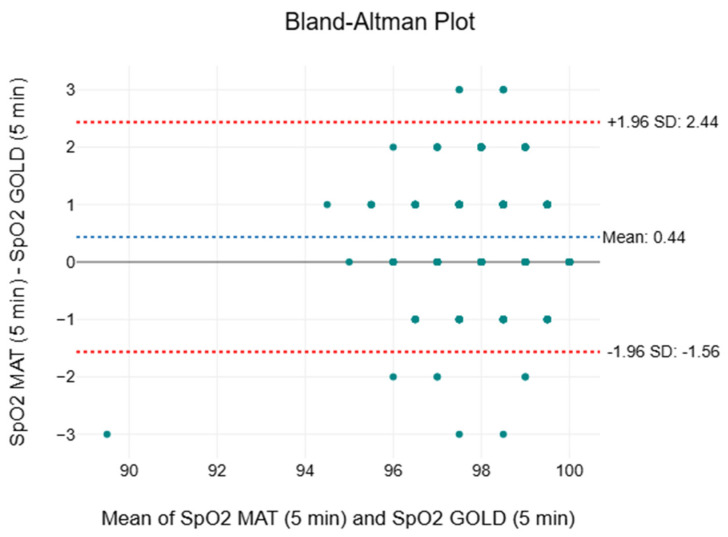

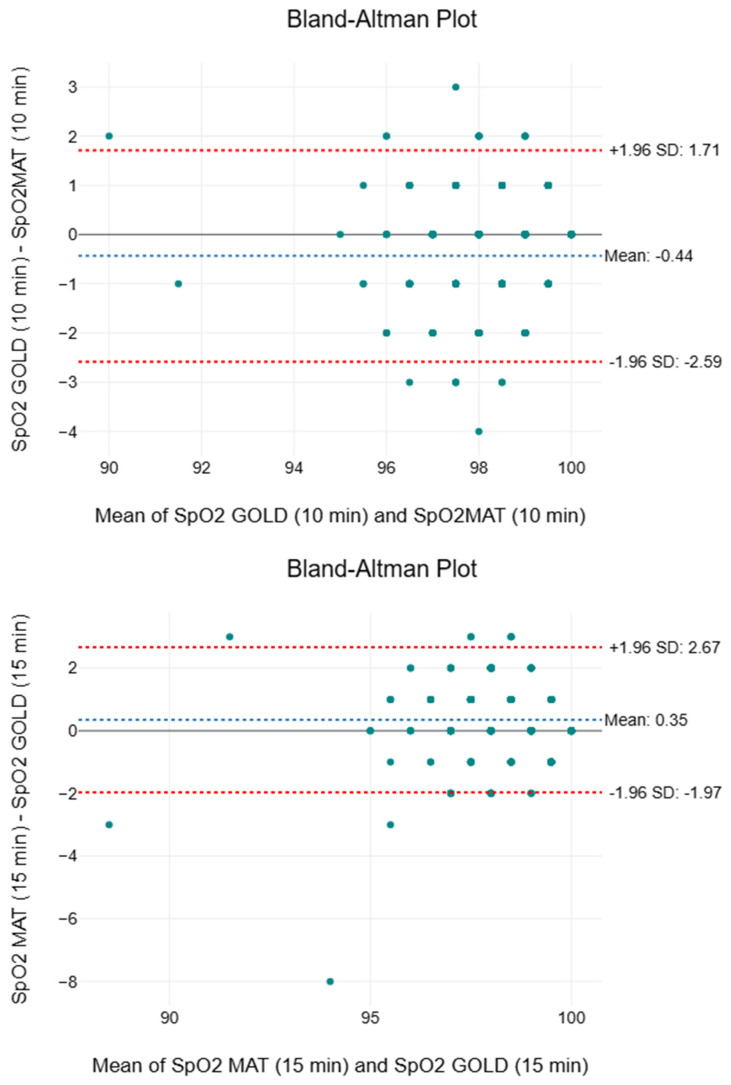
Compilation of Bland–Altman plots for oxygen saturation.

Table 7 outlines the descriptive and inferential statistics, including the two-way ANOVA, effect sizes, and MAPE, for SpO2. The means and medians recorded by both measurement methods ranged between 98 and 99% throughout. The IQR of the paired SMART MAT device (98 to 98%) were uniform and consistently overlapped with those of the clinical standard (97 to 99%). The F-values decreased with time from 58.7 to 28.5. Statistically significant differences (*p* < 0.001) were observed between the two measurement methods throughout. However, the effect sizes gradually reduced from 0.03 to 0.02. The MAPE values were consistently low, with values of 0.83%, 0.87%, and 0.89%, respectively. Figure 9 displays a compilation of the Bland–Altman plots for SpO2. The mean bias ranged from −0.44 to 0.44 mmHg, an almost insignificant variation between the two measurement methods. The 95% limits of agreement spanned from −2.59 to 2.67 mmHg.

## 4. Discussion

As the first large-scale validation study to establish the accuracy and validity of the SMART MAT, a novel vital sign monitoring device utilising multimode fibre optic technology, the findings of the study suggest that its performance is likely comparable to those of gold/clinical standard measurements. The similarities in the mean, median, and IQR of HR (Table 3) suggest minimal variability between the two devices. The consistency of the SMART MAT improves over time, as reflected in the convergence of its IQR with those of the gold standard, potentially due to ongoing calibration or system refinement. Although the first quartile values of the SMART MAT are 1 to 2 beats/min lower, this difference falls within the acceptable measurement bias of 10% or ±5 beats/min, as established by previous studies on novel wearable and contactless devices per the American National Standards Institute (ANSI) guidelines [54]. The non-statistically significant differences, negligible effect sizes, and low MAPE values that meet the acceptable standards for HR monitoring also suggest that the SMART MAT shows good agreement with the gold standard for HR measurement [55]. With reference to Figure 5, the majority of the plots fell within the 95% LOA, exhibiting a random scatter pattern with tight clustering around the mean bias close to zero. This indicates that despite exceeding the acceptable LOA of ±5 beats/min, the agreement between methods is likely acceptable in practical terms due to the observed consistency and minimal bias [54]. The SMART MAT is also validated against the clinical standard for monitoring RR, as evidenced by the similar mean and median, along with the identical IQR (Table 4), all of which adhere to the acceptable measurement bias of 10% or ±3 breaths/min [54]. While the upward trend in F-values and the corresponding decrease in *p*-values may signify a growing statistical divergence between the two measurement methods, the effect size remains negligible throughout, and the consistently low MAPE values continue to support the accuracy of the SMART MAT. Taken together, these findings suggest that, although final reading interval demonstrated statistical significance, the clinical implication of this difference is likely minimal. As shown in Figure 6, although the acceptable LOA of ±3 breaths/min was exceeded and one notable outlier in the third interval was observed, the mean bias remained negligible and the data exhibited random scatter without proportional bias [54]. Overall, the SMART MAT remains aligned with the acceptable clinical standard for RR measurement.

When evaluating the performance of the paired SMART MAT devices against the gold/clinical standard devices for BP and SpO2, it is important to note that these results do not reflect the inherent accuracy of the SMART MAT. As auxiliary components of the complete SMART MAT set-up, these paired devices utilise fundamentally different technologies for vital sign monitoring. The paired SMART MAT device consistently overestimated the mean, median, and IQR compared to the gold standard for SBP and DBP (Table 6 and Table 7). Notably, the SBP readings exceeded the acceptable bias and SD thresholds of ±5 and ±8 mmHg, respectively, as defined by the Advancement of Medical Instrumentation in a previous study evaluating the accuracy of continuous non-invasive arterial pressure monitoring devices [56]. The SD of the DBP readings also exceeded the acceptable threshold of ±8 mmHg throughout [56]. The relative inaccuracy of the SBP measurements is further affirmed by the exceptionally high F-values and statistically significant differences throughout. Conversely, the DBP readings showed a more positive trend of exhibiting improved accuracy over time based on the F-values and *p*-values. However, the small to negligible effect sizes and consistently low MAPE values for both SBP and DBP suggest that these differences may be clinically insignificant, with strong overall agreement with the gold standard. With reference to Figure 7 and Figure 8, while the upper and lower LOAs significantly deviated from the acceptable LOA of ±5 mmHg, a progressive narrowing of the LOA range as generally observed, suggesting improved agreement between the two methods over time [56]. Most plots fell within the 95% LOA and were randomly scattered, with SBP plots showing tighter clustering around the mean bias, indicating acceptable agreement between methods. When interpreted as a whole, the findings suggest that while the paired SMART MAT device shows potential for BP monitoring, its readings, particularly for SBP, should be interpreted with caution. With regard to SpO2, the identical mean and IQR, along with a near-identical median (Table 7), signify a high level of consistency and an absence of extreme deviations in measurements by the paired SMART MAT device when compared to the clinical standard. While the exceptionally high F-values and statistically significant differences may suggest variability between the devices, the consistently small effect sizes indicate that the statistical differences are unlikely to be clinically significant. This is particularly relevant for SpO2 in healthy adults, where the normal values typically range from 95 to 100%, and minor statistical deviations may not reflect meaningful clinical discrepancies [57]. Given the non-parametric nature of SpO2, many studies reference mean absolute error (MAE) values instead of MAPE. As recommended by the FDA and cited in previous studies, the acceptable MAE ranges between 2 to 3% [58,59,60]. Based on our raw data, the calculated MAE values of 0.99%, 1.02%, and 1.09%, respectively, fall within the acceptable range. As shown in Figure 9, with the exception of an outlier in the third interval, the consistently negligible mean bias, random scatter, and LOAs falling within the acceptable LOA of 3% indicate reasonable agreement between measurement methods [61]. Therefore, the findings support the potential validity of the paired SMART MAT device for SpO2 measurement.

While the SMART MAT demonstrates promising results in vital sign monitoring among healthy adults, its current applicability appears most be primarily suited to community and routine care settings. In the case of HR, most of the values fell within the normal range of 60 to 100 beats/min [62]. The accuracy of the SMART MAT in measuring HR beyond this range, therefore, should be applied with caution. Moreover, due to the highly sensitive nature of HR, governed by rapid autonomic and endocrine reflexes, fluctuations in cardiac rhythm and variability in response to physiological, psychological, and environmental changes were not captured in this study [63,64]. Regarding RR, previous research has shown it to be more sensitive than other vital signs in determining critically ill patients and serious adverse events [65]. Additionally, the performance of the paired SMART MAT device cannot be extrapolated to hypoxemic conditions with SpO2 levels below normal. Regulatory standards require SpO2 monitoring devices to undergo validation through controlled desaturation studies to ensure accuracy at low oxygen saturation levels [66,67]. Further testing is thus warranted before employing the SMART MAT in acute or clinical care settings. Lastly, although BP measurement using the paired SMART MAT device demonstrated the lowest accuracy among the vital signs assessed, the device is accepted and registered for home BP monitoring (see Instrumentation). To improve the overall performance of the SMART MAT system, a validated, clinical-grade BP monitor is recommended as a substitution for the current paired device to reduce measurement discrepancies, if deemed financially feasible [68].

The present study has several limitations. Most of the participants were young adults, with only five (1.56%) being aged 65 and above. This does not accurately reflect the typical hospitalised patient demographic and the elderly population in Singapore [69,70]. Its accuracy must be further validated through rigorous testing within clinical settings and on specialised populations. Vital sign measurements were conducted under controlled conditions, where participants were instructed to remain supine and still. These restrictions fail to reflect real-world conditions and limit real-world applicability, where movement, environmental factors, and improper device positioning may influence measurement accuracy. However, it is essential to note that such factors can affect all monitoring devices, which typically require time to recalibrate upon detecting movement. Based on observation, the SMART MAT requires approximately 5 to 10 s for recalibration following movement detection. Subsequent validation studies should evaluate the SMART MAT’s performance and ability to maintain signal integrity under dynamic conditions. Lastly, the long-term performance of the SMART MAT was not assessed in this study. As observed by the increasingly statistically significant differences between the SMART MAT and the clinical standard for RR over time in Table 4, the long-term performance of the SMART MAT technology remains uncertain. Future longitudinal research on the SMART MAT is recommended to evaluate the device’s performance and accuracy over extended periods of use before large-scale adoption.

## 5. Conclusions

The study demonstrates that the SMART MAT’s multimode optic fibre technology has demonstrated reasonable accuracy and validity in measuring vital signs, specifically HR and RR, among healthy adults in the community and routine care settings. While both paired SMART MAT devices measuring BP and SpO2 appear acceptable for these settings, the SBP showed reduced accuracy and should be interpreted with caution. To enhance the overall accuracy and reliability of the SMART MAT vital sign monitoring system, alternative paired BP devices should be investigated. Further rigorous validation studies are warranted to evaluate the accuracy of the SMART MAT against gold/clinical standard measurements in specialised and acute populations. Additionally, its long-term performance and performance under real-world conditions should be evaluated prior to wider implementation.

## 6. Patents

Chen ZH, Teo JT, Yang XF, inventors; Agency for Science, Technology and Research, assignee. A vital signs detecting device and a method for detecting vital signs [Internet]. Singapore patent 10201404690R. 2014 Oct 30. 34 p. Available from: https://digitalhub.ipos.gov.sg/FAMN/eservice/IP4SG/CM_UrlRewrite?p=f54d2c97-c492-42eb-9e86-c3bc9fd1a044, accessed on 21 June 2025

Zhu Y, Maniyeri J, Foo SF, Guan C, Zhang HH, Hao EJZ, Phua JE, Biswas J, inventors; Agency for Science, Technology and Research, assignee. Multi-channel ballistocardiography with cepstrum smoothing and quality-based dynamic channel selection [Internet]. Singapore patent 11201703737Q. 2017 Jun 29. 20 p. Available from: https://digitalhub.ipos.gov.sg/FAMN/eservice/IP4SG/CM_UrlRewrite?p=03e86a67-fe7a-4e89-b230-08f3c97e24e7, accessed on 21 June 2025).

## Figures and Tables

**Table 1:** Baseline demographic and anthropometric data of SMART MAT I + II.

Characteristics	Female (Mean ± SD)	Male (Mean ± SD)
Number	131	190
Age (years)	28.6 ± 11.9	28.2 ± 9.11
Height (cm)	161 ± 13.3	172 ± 6.72
Weight (kg)	55.9 ± 10.9	72.0 ± 12.9
Body mass index (kg/$m^{2}$)	21.3 ± 4.00	24.2 ± 3.55
Muscle mass (kg)	37.1 ± 3.56	54.7 ± 7.99
Body fat (%)	28.2 ± 7.87	20.0 ± 13.3
Total body water (%)	50.2 ± 4.76	56.1 ± 6.82

SD = standard deviation; cm = centimetre; kg = kilogram; m = metre.

**Table 2:** Two-way ANOVA between SMART MAT I and II.

Vital Sign	F	*p*	$n^{2}$
HR	0.07	0.791	<0.01
RR	3.79	0.052	0.01
SBP	1.60	0.207	<0.01
DBP	3.01	0.084	0.01
SpO2	0.74	0.392	<0.01

$n^{2}$ = effect size; HR = heart rate; RR = respiratory rate; SBP = systolic blood pressure; DBP = diastolic blood pressure; SpO2 = oxygen saturation.

**Table 3:** Descriptive and inferential statistics for heart rate.

Time Interval	Mean ± SD (bpm)	Median (bpm)	IQR (bpm)	F	*p*	$n^{2}$	MAPE (%)
3rd minute							
GOLD	71 ± 12	71	64 to 79	1.95	0.163	<0.01	6.45
SMART MAT	71 ± 12	71	62 to 79				
8th minute							
GOLD	71 ± 11	70	62 to 78	3.02	0.083	<0.01	6.24
SMART MAT	70 ± 12	71	61 to 78				
13th minute							
GOLD	70 ± 12	70	62 to 78	2.75	0.098	<0.01	6.44
SMART MAT	70 ± 12	70	61 to 77				

SD = standard deviation; bpm = beats per minute; IQR = interquartile range; $n^{2}$ = effect size; MAPE = mean absolute percentage error; GOLD = electrocardiogram.

**Table 4:** Descriptive and inferential statistics for respiratory rate.

Time Interval	Mean ± SD (bpm)	Median (bpm)	IQR (bpm)	F	*p*	$n^{2}$	MAPE (%)
5th minute							
CLINICAL	15 ± 4	15	13 to 18	0.02	0.892	<0.01	18.3
SMART MAT	15 ± 4	15	13 to 18				
10th minute							
CLINICAL	15 ± 4	15	13 to 18	3.70	0.055	<0.01	17.9
SMART MAT	16 ± 4	16	13 to 18				
15th minute							
CLINICAL	15 ± 4	15	13 to 18	5.47	0.020 *	<0.01	15.3
SMART MAT	16 ± 4	16	13 to 18				

SD = standard deviation; bpm = breaths per minute; IQR = interquartile range; $n^{2}$ = effect size; MAPE = mean absolute percentage error; CLINICAL = manual counting of respiratory rate; * *p* < 0.05.

**Table 5:** Descriptive and inferential statistics for systolic blood pressure.

Time Interval	Mean ± SD (mmHg)	Median (mmHg)	IQR (mmHg)	F	*p*	$n^{2}$	MAPE (%)
3rd minute							
GOLD	115 ± 15	113	106 to 123	87.7	<0.001 *	0.04	7.16
SMART MAT	118 ± 17	120	110 to 131				
8th minute							
GOLD	114 ± 14	112	104 to 122	72.9	<0.001 *	0.02	5.67
SMART MAT	118 ± 14	117	108 to 127				
13th minute							
GOLD	114 ± 14	111	103 to 121	74.5	<0.001 *	0.02	5.39
SMART MAT	118 ± 14	117	107 to 125				

SD = standard deviation; mmHg = millimetres of mercury; IQR = interquartile range; $n^{2}$ = effect size; MAPE = mean absolute percentage error; GOLD = sphygmomanometer; * *p* < 0.05.

**Table 6:** Descriptive and inferential statistics for diastolic blood pressure.

Time Interval	Mean ± SD (mmHg)	Median (mmHg)	IQR (mmHg)	F	*p*	$n^{2}$	MAPE (%)
3rd minute							
GOLD	68 ± 10	67	61 to 74	26.7	<0.001 *	0.01	9.22
SMART MAT	70 ± 10	69	63 to 77				
8th minute							
GOLD	68 ± 10	67	60 to 74	19.7	<0.001 *	0.01	8.76
SMART MAT	70 ± 10	69	63 to 76				
13th minute							
GOLD	68 ± 10	66	61 to 74	0.80	0.370	<0.01	8.78
SMART MAT	68 ± 9	67	62 to 74				

SD = standard deviation; mmHg = millimetres of mercury; IQR = interquartile range; $n^{2}$ = effect size; MAPE = mean absolute percentage error; GOLD = sphygmomanometer; * *p* < 0.05.

**Table 7:** Descriptive and inferential statistics for oxygen saturation.

Time Interval	Mean ± SD (%)	Median (%)	IQR (%)	F	*p*	$n^{2}$	MAPE (%)
5th minute							
CLINICAL	98 ± 1	98	97 to 99	58.7	<0.001 *	0.03	0.83
SMART MAT	98 ± 1	99	98 to 99				
10th minute							
CLINICAL	98 ± 1	98	97 to 99	51.0	<0.001 *	0.03	0.87
SMART MAT	98 ± 1	99	98 to 99				
15th minute							
CLINICAL	98 ± 1	98	97 to 99	28.5	<0.001 *	0.02	0.89
SMART MAT	98 ± 1	99	98 to 99				

SD = standard deviation; IQR = interquartile range; $n^{2}$ = effect size; MAPE = mean absolute percentage error; CLINICAL = NellcorTM pulse oximeter; * *p* < 0.05.

## Data Availability

The mini set data supporting the findings of this study are available at https://figshare.com/s/d85e63a9985d029f14e5 (accessed on 27 June 2025).

## Supplementary Materials

The following supporting information can be downloaded at: https://www.mdpi.com/article/10.3390/s25175321/s1, Supplementary File S1: Sensorgram Plots; Supplementary File S2: Comparative Analysis.

## Abbreviations

The following abbreviations are used in this manuscript:

HR	Heart rate
RR	Respiratory rate
SBP	Systolic blood pressure
DBP	Diastolic blood pressure
SpO2	Oxygen saturation
BP	Blood pressure
$n^{2}$	Effect size
MAPE	Mean Absolute Percentage Error
IQR	Interquartile range
ANSI	American National Standards Institute
MAE	Mean Absolute Error
