# Supplementary material for: SMART MAT: Fibre Optic Innovation for Bedside Monitoring and Validation of Continuous Vital Signs
Source: Sensors (Basel). 2025 Aug 27;25(17):5321. doi: 10.3390/s25175321 (PMC12431380; doi:10.3390/s25175321)

Supplementary File 1: Sensorgram Plots

The Python script uses Matplotlib to generate sensorgram plots for heart rate and respoiratory rate, comparing the mean values from the SMART MAT against gold/clinical standard measurements. Each plot shows data points at the specified time intervals, with solid lines for gold/clinical standards and dashed lines for SMART MAT measurements.

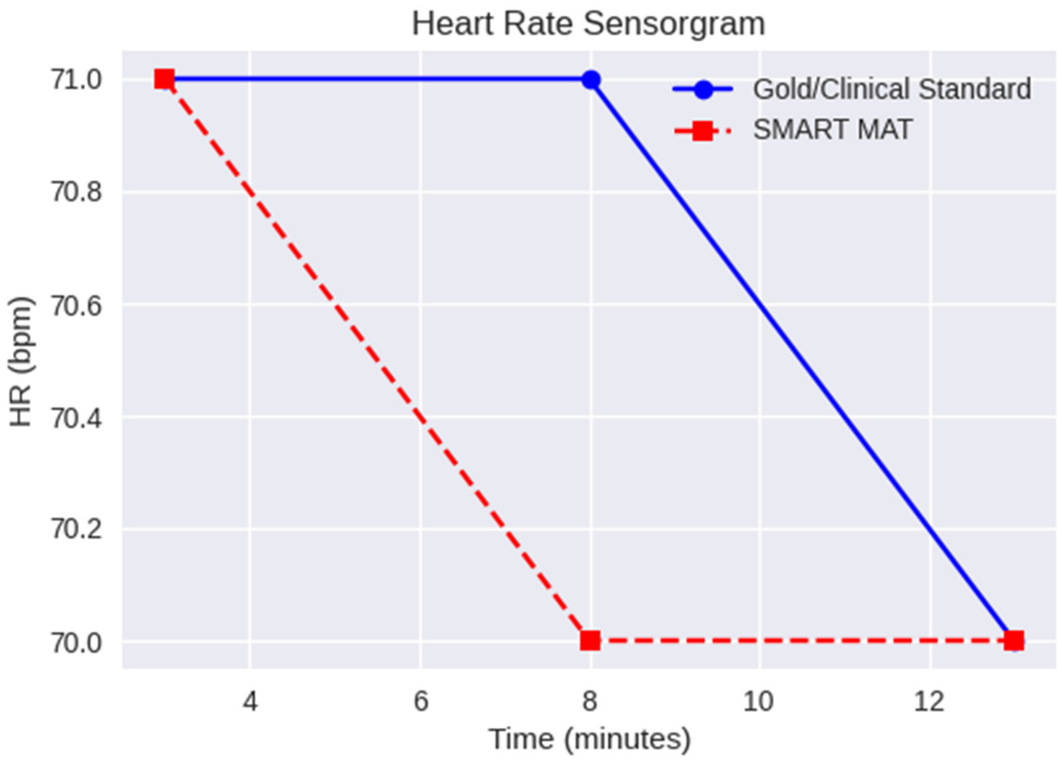

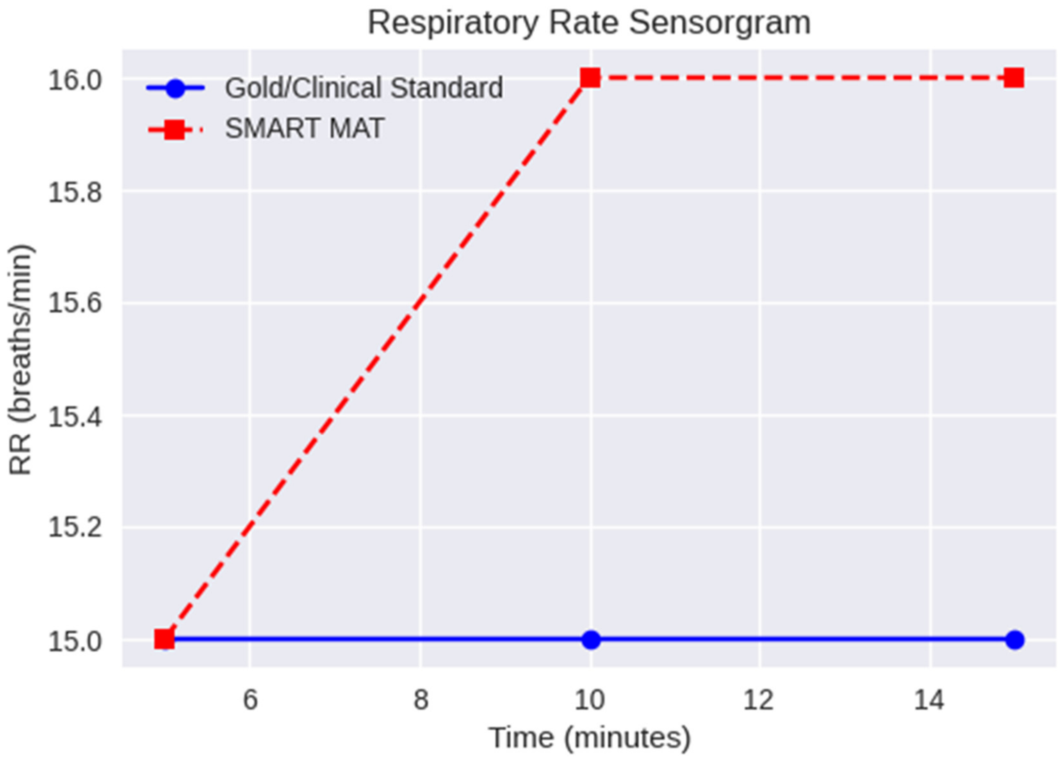

Supplement: Supplementary file 1 [file sensors-25-05321-s001.zip › Supplementary File S1.pdf]
