# Supplementary material for: SMART MAT: Fibre Optic Innovation for Bedside Monitoring and Validation of Continuous Vital Signs
Source: Sensors (Basel). 2025 Aug 27;25(17):5321. doi: 10.3390/s25175321 (PMC12431380; doi:10.3390/s25175321)
Supplement: Supplementary file 1 [file sensors-25-05321-s001.zip › Supplementary File S2.pdf]

Supplementary File 2: Comparative Analysis

The comparative analysis table compares the SMART MAT (including paired devices for BP and SpO2) with the gold/clinical standard devices used in the study. The gold/clinical standard devices are the Schiller CardioVit AT-1 G2 ECG (HR), manual counting (RR), Bokang LCD Mercury-Free Sphygmomanometer (BP), and Nellcor Portable SpO2 Monitor (SpO2).

The evaluation includes stability (via MAPE and p-values), drift (changes in mean values), response time (based on calibration and measurement times), and resolution (based on device specifications). The SMART MAT performs comparably to gold/clinical standards for HR and SpO2, with slight limitations in RR and SBP accuracy, as noted in the manuscript.

Parameter	Vital Sign	SMART MAT	Gold/Clinical Standard	Comments
**Stability**	HR	High: MAPE 6.24–6.45%, no significant differences ($p > 0.05$) across three intervals.	High: ECG provides stable, continuous HR measurements with minimal variability.	SMART MAT's fibre optic BCG-based detection maintains stability comparable to ECG, with consistent mean and IQR values.
	RR	Moderate: MAPE 15.3–18.3%, significant difference at 15th minute ($p = 0.020$).	High: Manual counting is highly stable but labor-intensive.	SMART MAT's RR detection shows slight variability, likely due to sensitivity to chest movement.
	SBP	Moderate: MAPE 5.39–7.16%, significant differences ($p < 0.001$) with higher readings.	High: Manual sphygmomanometer ensures stable readings via auscultatory method.	Paired BP device's oscillometric method may contribute to variability.
	DBP	High: MAPE 8.76–9.22%, significant differences only at first two intervals ($p < 0.001$).	High: Manual sphygmomanometer provides consistent DBP readings.	Paired BP device shows improving stability over time.
	SpO2	High: MAPE 0.83–0.89%, MAE 0.99–1.09% within FDA standards.	High: Nellcor monitor is robust under varying conditions.	Paired pulse oximeter maintains high stability, with near-identical means.
**Drift**	HR	Negligible: Mean values stable (70–71 bpm) across intervals.	Negligible: ECG drift is minimal due to direct electrical measurement.	SMART MAT's fibre optic system shows no significant drift.
	RR	Slight: Mean increases from 15 to 16 breaths/min, significant at 15th minute.	None: Manual counting eliminates drift.	Possible drift in SMART MAT due to calibration sensitivity over time.

Parameter	Vital Sign	SMART MAT	Gold/Clinical Standard	Comments
	SBP	Low: Mean stable at 118 mmHg, but consistently higher than gold standard.	None: Manual method avoids drift.	Paired device's consistent overestimation suggests systematic bias, not drift.
	DBP	Negligible: Mean stable (68–70 mmHg), no significant difference at 13th minute.	None: Manual method avoids drift.	Paired device shows minimal drift, improving over time.
	SpO2	Negligible: Mean stable at 98–99%, within 1% of clinical standard.	Negligible: Nellcor monitor designed for minimal drift.	Paired pulse oximeter shows no significant drift.
**Response Time**	HR	~5 seconds: Requires ~5–10 seconds for recalibration post-movement.	Near-instantaneous: ECG provides real-time HR data.	SMART MAT's response is slightly delayed due to signal processing.
	RR	~5–10 seconds: Detects chest displacement after calibration.	~60 seconds: Manual counting over 1 minute.	SMART MAT offers faster response for continuous monitoring.
	SBP	~30 seconds: Oscillometric method requires cuff inflation/deflation.	~30–60 seconds: Manual auscultatory method.	Comparable response time, but SMART MAT's automation reduces labor.
	DBP	~30 seconds: Same as SBP.	~30–60 seconds: Same as SBP.	Similar to SBP, automation improves efficiency.
	SpO2	~5 seconds: Photoelectric detection provides rapid readings.	~5 seconds: Nellcor monitor offers near-instantaneous readings.	Comparable response time, both using similar pulse oximetry technology.
**Resolution**	HR	1 bpm: Based on BCG signal processing.	1 bpm: ECG standard resolution.	Equivalent resolution suitable for clinical use.
	RR	1 breath/min: Based on pressure variation detection.	1 breath/min: Manual counting resolution.	Equivalent resolution, adequate for routine monitoring.
	SBP	1 mmHg: Oscillometric device resolution.	1 mmHg: Sphygmomanometer resolution.	Equivalent resolution, but accuracy lower in SMART MAT's paired device.
	DBP	1 mmHg: Same as SBP.	1 mmHg: Same as SBP.	Equivalent resolution, with improving accuracy over time.

Parameter	Vital Sign	SMART MAT	Gold/Clinical Standard	Comments
	SpO2	1%: Photoelectric oximeter resolution.	1%: Nellcor monitor resolution.	Equivalent resolution, with high accuracy in both.
